# Effects of microbial inoculants and planting density on soybean summer-sown growth, nutrients accumulation and yield in Southern Xinjiang

**DOI:** 10.3389/fpls.2026.1835826

**Published:** 2026-06-10

**Authors:** Wenya Wang, Mengyang Li, Kuo Li, Xinhao Li, Jiahao Liu, Tingyong Mao, Xiaogang Yin, Long Ma, Desheng Wang, Lili Yang, Yunlong Zhai

**Affiliations:** 1 College of Agriculture, Tarim University, Alar, China; 2 Key Laboratory of Genetic Improvement and Efficient Production for Specialty Crops in Arid Southern Xinjiang of Xinjiang Corps, Alar, China; 3 College of Agronomy and Biotechnology, China Agricultural University, Beijing, China

**Keywords:** Bacillus, crop growth, Glycine max, plant development, planting density, rhizobia

## Abstract

**Introduction:**

Soybean cultivation in southern Xinjiang of China is constrained by multiple natural and anthropogenic factors. This study explored the synergistic effects of microbial inoculation and planting density on summer-sown soybeans to find the optimal solution for high and stable yield.

**Methods:**

Herein, A two-year field experiment was conducted with a split-plot design. Four planting densities were applied in the main plots: 123, 000 plants hm^–2^ (D1); 82, 000 plants hm^–2^ (D2); 59, 000 plants hm^–2^ (D3); and 44, 000 plants hm^–2^ (D4) . In the subplots, eight inoculation treatments were established: no inoculation (C0, control); single inoculation with Bacillus (T0); co-inoculation with *Bacillus* and *Rhizobium fredii* SMH12 (T1), *Bacillus* and *Rhizobium fredii* SN7-2 (T2), Bacillus and and Rhizobium fredii HN01 (T3); and single inoculation with *Rhizobium fredii* SMH12 (T4), *Rhizobium fredii* SN7-2 (T5), and *Rhizobium fredii* HN01 (T6).

**Results:**

The results showed that microbial inoculation and planting density significantly affected plant growth, root nodulation, nutrient accumulation, and yield. T2 resulted in the highest yield under the D3 density (5174 .59–5385. 67 kg hm^–2^), which was improved by 7 .8 –69.9% over that of other treatments. This yield improvement was achieved mainly through increasing seed number per plant and 100-seed weight without reducing pod number per plant. The increase in 100-seed weight was related to higher root nodulation potential and nutrient accumulation level. The D3 density optimized plant morphology (especially in increasing main-stem node number and stem diameter), thereby contributing to higher yield. Correlation analysis showed that yield was highly positively correlated with main-stem node number and root nitrogen accumulation. Principal component analysis identified D3T2 as the largest contributor to plant growth (comprehensive score F = 1 . 28).

**Conclusion:**

Overall, These findings suggest that co-inoculation with *Bacillus* and *Rhizobium* SN7–2 under a planting density of 59, 000 plants hm^–2^ is suitable for high-yield cultivation of summer-sown soybeans under the experimental conditions. This study provides an effective agronomic strategy for enhancing soybean yield potential and stress resistance in arid regions with poor soil fertility.

## 1 Introduction

Soybean (*Glycine max* L.) is widely cultivated in the world as a critical raw material for vegetable oil, protein-rich human food, and animal feed (Song et al., 2016). Since the 1970s, the expansion of soybean cultivation has outpaced that of all other major crops. Nowadays, soybean-growing areas account for approximately 6% of the global arable land (Zhao et al., 2023). Genetically modified soybeans cultivated in the United States and Argentina exhibit superior yield performance, despite their relatively low protein content. Non-genetically modified soybeans grown in China have elevated protein content, yet lack yield advantages with inferior productivity. As global demand for soybeans continues to rise, expanding their growing area is crucial for the maintenance of national food and oil security (Mauser et al., 2015; Xu et al., 2022; Wang et al., 2023). In Xinjiang of China, the soybean yield reached a record high of 6.8 tonnes in 2020 under a planting density of 300, 000 plants hm^–2^ (Ran et al., 2023). This region experiences substantial diurnal temperature fluctuations and intense solar radiation. The environmental conditions are conducive to farm crops growth, indicating considerable development potential (Yang et al., 2024). However, Xinjiang is an arid region with relatively poor soil quality and limited water and nutrient supply capacity.local soybean cultivation practices remain relatively extensive, resulting in low unit-area yields and high production costs. These factors impede the sustainable development of soybean production in the Xinjiang region.

Soybeans are legumes capable of biological nitrogen fixation through symbiosis with root-nodulating rhizobia, thus requiring lower nitrogen inputs than cereal crops (De Souza Júnior et al., 2026). High-quality rhizobial inoculants can enhance nitrogen fixation, improve soybean yield and quality, and reduce fertilizer requirement, achieving cost savings and production efficiency gains (Alves et al., 2003; Herridge et al., 2008; Ohwaki and Sugahara, 1997). Rhizobia, a group of nitrogen-fixing bacteria, are able to survive saprophytically in the soil or form symbiosis with leguminous host plants (Xia et al., 2026). Once established in root nodules, rhizobia convert atmospheric nitrogen into a form utilizable by the host plant, which in turn provides photosynthetically derived carbon to support rhizobial metabolism (Kaschuk et al., 2009; Taylor and Menge, 2018). The legume-rhizobium symbiosis represents a classic model of plant-microbe mutualism, conferring benefits to host plants and mediating the global nitrogen cycle (Coskun et al., 2017).

*Bacillus* spp. are a common type of plant growth-promoting rhizobacteria (PGPR) that colonize plant roots (Zhou et al., 2023). By establishing mutually beneficial relationships with host plants, PGPR promote plant growth primarily through the modification of root morphology, physiology, and function (Wang et al., 2025). This could mitigate impairments to the absorption and utilization of water and minerals by plants (Mohanty et al., 2021). PGPR colonization has been shown to increase root surface area and stimulate root cell division in wheat (Levanony and Bashan, 1989). The use of PGPR in maize improves the diameter and length of lateral roots (Dos Santos et al., 2020). Additionally, PGPR inoculation facilitates the development of root hairs and cortical tissues in tomato (Jensen et al., 2024). *Bacillus* strains, as typical PGPR and excellent microbial inoculants, are widely used to promote plant growth and suppress soil-borne diseases (Abdelmoteleb et al., 2022). However, distinct plant growth-promoting properties of various microorganisms limit the ability of single strains to deliver ideal effects. Thus, achieving yield improvements with single-strain inoculants is challenging (Timofeeva et al., 2024). *Bacillus* strains are increasingly used in the development of rhizobium-based co-inoculants. Such co-inoculants can prominently enhance the capacity of biological nitrogen fixation in leguminous plants, promoting their growth and development (Zhou et al., 2016).

Microbial co-inoculants containing two or more non-antagonistic strains offer multifaceted functional benefits and enhanced plant growth-promoting effects (Zhang et al., 2023). Co-inoculation with *Pseudomonas fluorescens* (a phytohormone producer) and *Azospirillum brasilense* (a diazotrophic bacterium) improved the harvest index (16%) and grain yield (20%) of rice (*Oryza sativa*) under field conditions (Jack et al., 2021). For soybean, strategic application of *Bradyrhizobium japonicum* with *Bacillus aryabhattai* and *Paenibacillus mucilaginosus* increased the levels of nitrogen (20%) and phosphorus (12%) in the rhizosphere soil (Xing et al., 2022). In addition to soil nutrient availability, plant stress tolerance was enhanced following co-application of different rhizobia or PGPR with rhizobia, which stimulated crop growth (Jesus et al., 2018; Jabborova et al., 2020). Plants co-inoculated with *Pseudomonas chlororaphis* and *Bacillus altitudinis* exhibited notable increases in total height (24%) and stem diameter (34%) (Zhang et al., 2023). Thus, harnessing microbial co-inoculants to optimize the balance between shoot dry matter accumulation and root nutrient acquisition in soybeans can alleviate inter-root competition for nitrogen, which supports high-yield potential.

Increasing planting density—a critical factor that shapes plant population structure—is the most direct strategy for improving soybean yield (Fang et al., 2026). Optimizing planting density can improve resource utilization efficiency in farmland (Bigatton et al., 2024) and enhance dry matter accumulation in crops (Guo et al., 2021). This strategy contributes to an increased number of effective pod density (Zheng et al., 2021), while improving crop harvest index, biomass, and yield (Zhang et al., 2021). For example, Friedman (2024) ameliorated field light conditions and nutrient supply status by optimizing planting density. A canopy structure was established to enhance population yield efficiency, resulting in a 14–30% increase in soybean biomass. However, inappropriate planting density often impedes crop growth and yield production. When row spacing is too narrow, intensified intraspecific competition for resources leads to a decreased number of pods per plant (Egli, 2022). Under high-density planting, pod yield per unit area is greater than under low-density planting, and yield increases with higher planting densities until a density of 3150, 000 plants hm^–2^ is reached for soybeans (Xu et al., 2021).

While the number of soybean plants per unit area and row spacing affect field biomass yield, cowpea (*Vigna unguiculata*) produces higher forage yield and lower seed weight with increasing plant density (Kamara et al., 2018; Loha et al., 2023; Luca and Hungría, 2014; Matsuo et al., 2018). Excessively high planting density inhibits plant growth and development, elevating the risk of lodging (Duan et al., 2023; Zhang et al., 2022). Under increased planting density, main stem and internodes are elongated in rice, while tiller growth and number are reduced in wheat (*Triticum aestivum*) (Hayashi et al., 2006; Nakano et al., 2012). High planting density alters the canopy structure of paper mulberry, intensifying competition for nutrients, light, and other resources (Clerget et al., 2016). As a consequence, crop photosynthesis is weakened, which increases the risk of pests and diseases, leading to yield decline (Testa et al., 2016; Xue et al., 2017). Choosing an appropriate planting density can adjust plant population structure, enhance resource utilization efficiency, and promote high-quality and high-yield crop production.

Soybean is one of the major crops produced in southern Xinjiang, where unique natural conditions and abundant sunlight resources provide high-yield potential. Crop planting densities in this region tend to be higher than those in other regions, but there is a paucity of research on the optimal planting density for soybeans. Microbial inoculation and planting density optimization are widely recognized as effective agronomic practices; however, their interactive effects on soybean growth, development, nutrient uptake, and yield in southern Xinjiang are still insufficiently explored. In this study, we hypothesized that enhancing nutrient uptake in the belowground parts of soybeans with different microbial inoculants can (1) boost nitrogen fixation by facilitating root nodulation; and (2) improve population yield by promoting plant growth and development under high-density planting. The objectives of this study were to (1) determine the yield improvement potential of soybean populations under different planting densities; (2) compare the effects of inoculating *Bacillus* and rhizobia on plant traits, nodulation characteristics, and nutrient accumulation under the same planting densities; and (3) elucidate the mechanisms by which different microbial inoculants and planting densities affect the yield of summer-sown soybeans in southern Xinjiang.

## 2 Materials and methods

### 2.1 Experimental site

The experiment was conducted from June 2021 to October 2022 at the Agricultural Experiment Station of Tarim University (Alar, Xinjiang, China), located on the northern edge of the Tarim Basin. Winter wheat is the immediately preceding crop cultivated at the experimental site. The ≥10 °C annual effective accumulated temperature is 4113 °C and the frost-free period lasts 220 days. The average daily sunshine duration is 9.5 hours from April to October, with longer daylight hours compared to inland regions. The average annual precipitation is approximately 50 mm, indicating scarce rainfall, characteristic of a warm temperate continental arid desert climate. The soil texture is loam, containing 7.89 g kg^–1^ of organic matter. The soil available phosphorus and potassium contents are 19.1 and 114 mg kg^–1^, respectively. The alkaline hydrolyzable nitrogen content of the soil is 33.6 mg kg^–1^ and the pH value is 7.9. The soil properties were determined based on methods described by Alves et al. (2019); Johnson et al. (2007), and Weidhuner et al. (2026). For summer-sown soybeans throughout the entire growth period, topdressing applications of 208.6 kg/hm² of urea, 119.2 kg/hm² of monoammonium phosphate, and 298 kg/hm² of potassium sulfate are required.

### 2.2 Experimental materials

The soybean variety used in the experiment was Jiyu 202. Soybean seeds were purchased from Alar City, Xinjiang Province, China. A PGPR strain (*Bacillus*) and three rhizobial strains (*Rhizobium fredii SMH12*, *R. fredii SN7-2*, and *R. fredii* HN01) were used as microbial inoculants. All strains were provided by the State Key Laboratory of Agricultural Microbiology at Huazhong Agricultural University (Wuhan, Hubei, China).

### 2.3 Experimental design

The experiment used a split-plot design with two factors. In the main plots, four different planting densities were applied: 123, 000 plants hm^–2^ (D1); 82, 000 plants hm^–2^ (D2); 59, 000 plants hm^–2^ (D3); and 44, 000 plants hm^–2^ (D4). In the subplots, single-strain and co-inoculants were used to establish eight treatments: water with no inoculant (C0, control); *Bacillus* + water (T0); *Bacillus* + *Rhizobium fredii* SMH12 (T1); *Bacillus* + *Rhizobium fredii* SN7-2 (T2); *Bacillus* + *Rhizobium fredii* HN01 (T3); *R.fredi* SMH12 + water (T4); *R.fredi* SN7-2 + water (T5); and *R.fredi* HN01 + water (T6). Microbial inoculation was implemented through seed coating. Briefly, 1 kg of soybean seeds were thoroughly mixed with 15 mL of each inoculant. The seeds were evenly coated and then dried in a cool and well-ventilated place before being manually sown on the same day. There were 32 treatments in total, with three replicates per treatment. The plot size was 4.5 m × 2 m each. The field management measures were identical to local agricultural practices.

### 2.4 Sampling and testing

1. Nodulation Characteristics: The nodulation status of soybean roots was assessed at the R6 (seed filling) stage. Ten representative plants with uniform growth were randomly selected from each treatment. The roots were completely excavated using a root auger to minimize damage. After cutting plants at the cotyledonary node, the roots were placed in nylon mesh bags and soaked in tap water for 30 minutes to loosen adhering soil. The roots were then gently rinsed with deionized water to remove residual soil, and excess moisture was removed using absorbent paper. All nodules were collected from the roots of each plant, and the total nodule number per plant was recorded. The fresh weight of nodules per plant was measured using a precision electronic balance. Subsequently, the nodules were heat-inactivated at 105 °C for 30 minutes and oven-dried at 80 °C until constant weight to determine dry weight (Stanisławska-Glubiak et al., 2021).
2. Plant Agronomic Traits: At the V4 (four node) stage of soybean, 10 representative plants with uniform growth were randomly selected from each treatment and labeled. Growth measurements (plant height and stem diameter) were conducted on the selected plants at the V4 (fourth node), R2 (full bloom), R4 (full pod), R6 (seed filling), and R8 (full maturity) stages. Plant height was measured from the cotyledonary node to the natural apex using a calibrated measuring tape. Stem diameter was quantified as the diameter of the second internode of the main stem (counted from the cotyledonary node) using a digital vernier caliper. The total number of main-stem nodes (from the cotyledonary node to the apex) was recorded for each plant (Osman et al., 2021).
3. Dry Matter Accumulation and Partitioning: Ten representative soybean plants with uniform growth were randomly selected from each treatment for destructive sampling at key growth stages. Plant samples were separated into different fractions as follows: stem and leaf at the R2 (full bloom) stage; root, stem, leaf, and pod at the R4 (full pod) and R6 (seed filling) stages; and stem, pod shell, and seed at the R8 (full maturity) stage. All plant fractions were placed in labeled paper envelopes, heat-inactivated at 105 °C for 30 minutes, and then oven-dried at 80 °C until constant weight. The dry weight of each fraction was measured using a precision balance, and the results were used to calculate per-plant dry matter accumulation and dry matter partitioning among organs. The dry matter partitioning ratio (%) was computed as follows: (Dry weight of a specific organ at the target stage/Total aboveground dry matter weight at the same stage) × 100 (Tourino et al., 2002; Xu et al., 2021).
4. Yield and Yield Components: At the R8 (full maturity) stage, one 1 m^3^ quadrat was selected from the center of each experimental plot for actual harvest and yield estimation. Ten representative soybean plants with uniform growth were randomly selected from each quadrat for laboratory-based measurements. The following yield components were quantified: pod number per plant, seed number per plant, seed weight per plant, and 100-seed weight. Seeds were adjusted to 13.5% moisture content by drying prior to weight measurement (De Souza Júnior et al., 2026).
5. Plant Nutrient Accumulation: Root samples were dried and pulverized before nutrient analysis. Total nitrogen, phosphorus, and potassium contents in dried and finely ground leaf samples were quantified using methods described by Jackson et al. (1991) and Mahajan et al. (2021).

### 2.5 Statistical analysis

Data were processed using Excel 2021 (Microsoft Corp., Redmond, WA, USA) and subjected to normality test prior to analysis. Two-way analysis of variance was conducted using IBM SPSS Statistics 27.0 (IBM Corp., Armonk, NY, USA) at a significance level of α = 0.05. pearson correlation analysis and principal component analysis (PCA) were performed following the steps described by Greenacre et al. (2022). Graphs were created using Origin 2024 (OriginLab Corp., Northampton, MA, USA).

## 3 Results

### 3.1 Plant growth responses to microbial inoculation and planting density

The plant height of soybeans showed accelerated growth rates from the four-node stage to the full-podding stage; subsequently, the growth rates slowed down after the seed-filling stage (**Table 1**). At the maturity stage, different effects of microbial inoculants emerged on plant height. An increase in planting density resulted in higher plant height. Under the high density (D1), plant height was highest in the T6 treatment with single-strain inoculant. Under the low density (D4), the T1 treatment with co-inoculant achieved the highest plant height. Under the medium densities of (D2, D3), plant height reached its peak in the T3 treatment, followed by the T4 treatment; the plant height in the T2 treatment with co-inoculant was relatively low, indicating effective inhibition of excessive plant growth.

**Table 1:** Effects of different treatments on plant height of summer-sown soybeans (cm).

Treatment	Four-node	Full bloom	Full pod	Full seed	Full maturity
Density (D)					
D1	13.793a	37.589a	54.098a	56.948a	59.776a
D2	13.335b	36.153b	51.756b	55.592b	57.474b
D3	12.532c	34.129c	49.762c	54.041c	55.295c
D4	12.116d	33.049c	47.911d	52.324d	54.139d
Inoculant (T)					
C0	13.405b	33.929b	48.791f	53.841bc	56.145a
T0	12.874cd	34.414b	49.62ef	54.291bc	56.365a
T1	12.65d	35.341a	50.305de	55.06ab	56.623a
T2	11.835e	34.51b	51.072cd	53.055c	55.865a
T3	13.215bc	35.915a	52.792ab	55.743ab	57.495a
T4	13.816a	35.854a	53.057a	56.736a	57.68a
T5	13.201bc	35.924a	49.455ef	54.129bc	56.202a
T6	12.556d	35.955a	51.96bc	54.956ab	56.993a
Interaction (D×T)					
D1C0	14.215abc	36.465cdefghi	54.315abc	56.585abcd	61.94ab
D1T0	13.55cdef	37.16abcdef	52.69bcde	56.89abcd	59.44abcd
D1T1	13.49cdef	37.58abcde	53.9abc	57abcd	57.73bcdefg
D1T2	12.89fghij	36.93abcdefg	54.59ab	55.19bcdefgh	59.96abc
D1T3	14.35ab	38.58a	55.25a	57.23abc	58.99abcde
D1T4	14.57a	37.79abcd	55.125a	59.425a	58.45abcdef
D1T5	14.12abcd	37.94abc	52.785bcde	56.635abcd	59.08abcde
D1T6	13.16efgh	38.27ab	54.13abc	56.63abcd	62.62a
D2C0	13.58cdef	35.83efghij	49.27ghij	55.06bcdefghi	57.84bcdefg
D2T0	13.075efghi	34.765ijklm	50.505efgh	55.045bcdefghi	57.68bcdefg
D2T1	13.34efg	37.04abcdefg	52.09cdef	56.18abcde	57.28cdefg
D2T2	12.29jkl	35.58fghij	51.53defg	54.01bcdefghij	56.61cdefgh
D2T3	13.54cdef	36.05defghi	53.49abcd	56.55abcd	59.5abcd
D2T4	14.16abcd	36.615bcdefgh	53.925abc	57.57ab	59.47abcd
D2T5	13.7bcde	37.165abcdef	50.625efgh	55.105bcdefgh	55.77cdefgh
D2T6	12.995efghij	36.18cdefghi	52.615bcde	55.22bcdefgh	55.64cdefgh
D3C0	13.43defg	32.08nop	46.06klm	52.48efghij	52.44h
D3T0	12.58hijk	33.765klmn	49.025hij	53.36cdefghij	55.8cdefgh
D3T1	12.08kl	33.68klmno	49.57ghij	54.77bcdefghi	55.6cdefgh
D3T2	11.22mn	33.18mno	50.13fghi	52.34efghij	53.52gh
D3T3	12.58hijk	35.27ghijk	52.69bcde	55.41bcdefg	56.58cdefgh
D3T4	13.575cdef	34.805ijklm	52.11cdef	56.04abcdef	57.3cdefg
D3T5	12.7ghijk	35.145hijkl	47.84jkl	53.28defghij	55.48cdefgh
D3T6	12.09kl	35.105hijkl	50.67efgh	54.65bcdefghi	55.64cdefgh
D4C0	12.395ijkl	31.34p	45.52m	51.24ij	52.36h
D4T0	12.29jkl	31.965op	46.26klm	51.87ghij	52.54h
D4T1	11.69lm	33.065mnop	45.66lm	52.29fghij	55.88cdefgh
D4T2	10.94n	32.35nop	48.04ijk	50.68j	53.37gh
D4T3	12.39ijkl	33.76klmn	49.74ghij	53.78bcdefghij	54.91defgh
D4T4	12.96efghij	34.205jklm	51.07efgh	53.91bcdefghij	55.5cdefgh
D4T5	12.285jkl	33.445lmno	46.57klm	51.495hij	54.48efgh
D4T6	11.98kl	34.265jklm	50.425efgh	53.325defghij	54.07fgh

D1, high-density planting at 123, 000 plants hm^–2^; D2 and D3, medium-density planting at 82, 000 and 59, 000 plants hm^–2^, respectively; D4, low-density planting at 44, 000 plants hm^–2^.

C0: non-inoculated control; T0, single inoculation with *Bacillus*; T1–T3, co-inoculation with *Bacillus* and *Rhizobium* SMH12, *Rhizobium* SN7-2, and *Rhizobium* HN01, respectively; T4–T6, single inoculation with *Rhizobium* SMH12, *Rhizobium* SN7-2, and *Rhizobium* HN01, respectively.

Data are presented as mean values (*n =* 5), and different lowercase letters in a column denote significant differences among the treatment groups (*p* < 0.05).

Under the same planting densities, T1 significantly increased stem diameter in soybeans compared to other treatments (**Table 2**). When D1 was used, the largest stem diameter was observed in the T1 treatment at the pod-filling stage. There was no significant difference in stem diameter between the T1 and T2 treatments, indicating that both co-inoculants alleviated the inhibitory effect of high planting density on stem diameter growth. Under the D3 density, the T1 treatment showed significantly larger stem diameter than other treatments (T1 > T2 > T0 > T3 > T6 > T5 > C0 > T4). With the same microbial inoculants, the stem diameter under the D4 density was significantly larger than that under other densities (by 5–20%).

**Table 2:** Effects of different treatments on stem diameter of summer-sown soybeans (cm).

Treatment	Four-node	Full bloom	Full pod	Full seed	Full maturity
Density (D)					
D1	0.344bc	0.388c	0.443c	0.49d	0.516d
D2	0.348b	0.412b	0.481b	0.535c	0.573c
D3	0.341c	0.405b	0.487b	0.557b	0.594b
D4	0.361a	0.43a	0.506a	0.583a	0.624a
Inoculant (T)					
C0	0.34a	0.396b	0.468bc	0.509d	0.541cd
T0	0.357a	0.407b	0.477bc	0.559b	0.597b
T1	0.359a	0.448a	0.535a	0.609a	0.641a
T2	0.35a	0.441a	0.524a	0.575b	0.606b
T3	0.35a	0.412b	0.484b	0.548bc	0.594b
T4	0.349a	0.388b	0.451bc	0.497d	0.525d
T5	0.34a	0.384b	0.443c	0.512d	0.549cd
T6	0.342a	0.394b	0.454bc	0.522cd	0.563c
Interaction (D×T)					
D1C0	0.32fgh	0.356gh	0.428fghij	0.456j	0.472m
D1T0	0.343bcdefgh	0.393bcdefgh	0.451defghij	0.5defghij	0.538hijkl
D1T1	0.401a	0.449abc	0.502bcdefg	0.549bcdefg	0.581efghi
D1T2	0.373abcde	0.456ab	0.498bcdefgh	0.535bcdefghi	0.558ghijk
D1T3	0.324efgh	0.376defgh	0.432fghij	0.467ij	0.491lm
D1T4	0.333defgh	0.35h	0.419hij	0.484ghij	0.512jklm
D1T5	0.317fgh	0.356gh	0.403j	0.458j	0.484lm
D1T6	0.343bcdefgh	0.37efgh	0.414ij	0.472hij	0.489lm
D2C0	0.333defgh	0.389cdefgh	0.454defghij	0.495efghij	0.544hijkl
D2T0	0.389abc	0.454ab	0.537abc	0.595b	0.626bcde
D2T1	0.347bcdefgh	0.442abc	0.5bcdefg	0.565bcde	0.607cdefg
D2T2	0.391ab	0.45abc	0.515bcde	0.582bc	0.61cdefg
D2T3	0.343bcdefgh	0.414abcdefgh	0.493bcdefghi	0.544bcdefg	0.595cdefgh
D2T4	0.327efgh	0.367fgh	0.446efghij	0.481ghij	0.501klm
D2T5	0.312gh	0.376defgh	0.481bcdefghij	0.511defghij	0.54hijkl
D2T6	0.34cdefgh	0.402abcdefgh	0.426ghij	0.507defghij	0.564fghij
D3C0	0.324efgh	0.403abcdefgh	0.482bcdefghij	0.519cdefghij	0.559fghijk
D3T0	0.317fgh	0.366fgh	0.465bcdefghij	0.558bcdef	0.608cdefg
D3T1	0.347bcdefgh	0.441abc	0.531abcd	0.665a	0.697a
D3T2	0.303h	0.414abcdefgh	0.545ab	0.595b	0.62bcdef
D3T3	0.38abcd	0.433abcde	0.493bcdefghi	0.59b	0.639abcde
D3T4	0.343bcdefgh	0.394bcdefgh	0.458cdefghij	0.498defghij	0.528ijklm
D3T5	0.368abcdef	0.398abcdefgh	0.454defghij	0.493fghij	0.517jklm
D3T6	0.345bcdefgh	0.389cdefgh	0.468bcdefghij	0.541bcdefgh	0.587defghi
D4C0	0.384abcd	0.437abcd	0.509bcdef	0.564bcde	0.589defghi
D4T0	0.38abcd	0.412abcdefgh	0.456cdefghij	0.586bc	0.614cdefg
D4T1	0.34bcdefgh	0.46a	0.605a	0.655a	0.679ab
D4T2	0.333defgh	0.445abc	0.536abc	0.587bc	0.636bcde
D4T3	0.354abcdefgh	0.425abcdef	0.52bcde	0.591b	0.649abcd
D4T4	0.391ab	0.44abc	0.484bcdefghij	0.526bcdefghij	0.559fghijk
D4T5	0.363abcdefg	0.405abcdefgh	0.433fghij	0.585bc	0.655abc
D4T6	0.34bcdefgh	0.417abcdefg	0.506bcdefg	0.567bcd	0.612cdefg

All treatments are defined in **Table 1** footnote.

Data are presented as mean values (*n =* 5), and different lowercase letters in a column denote significant differences among the treatment groups (*p* < 0.05).

**Figure 1:**
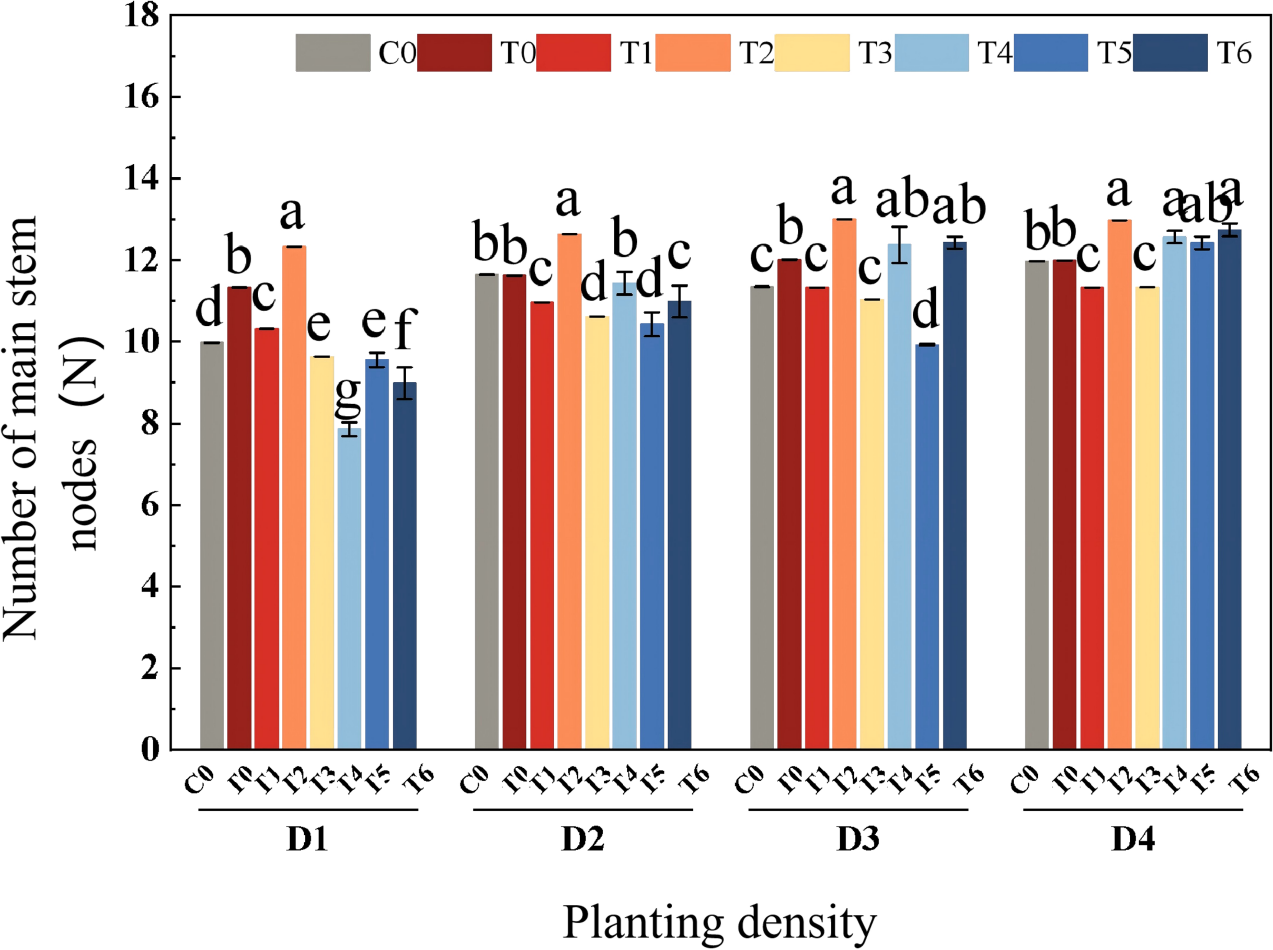
The number of main-stem nodes in summer-sown soybeans with different microbial inoculants and planting densities. D1, high-density planting at 123, 000 plants hm^–2^; D2 and D3, medium-density planting at 82, 000 and 59, 000 plants hm^–2^, respectively; D4, low-density planting at 44, 000 plants hm^–2^. C0: non-inoculated control; T0, single inoculation with *Bacillus*; T1–T3, co-inoculation with *Bacillus* and *Rhizobium* SMH12, *Rhizobium* SN7-2, and *Rhizobium* HN01, respectively; T4–T6, single inoculation with *Rhizobium* SMH12, *Rhizobium* SN7-2, and *Rhizobium* HN01, respectively. Error bars represent standard deviation of the mean (*n =* 5), and significant differences among the treatment groups are marked by different lowercase letters above the error bars (*p* < 0.05).

T2 was superior to other treatments in improving the number of main-stem nodes in soybeans under the same planting densities (by 8.5–20.3%) (**Figure 1**). When the same microbial inoculants were used, the number of main-stem nodes decreased with increasing planting density (D4 > D3 > D2 > D1). Among all treatments, D3T2 showed the greatest effect on the number of main-stem nodes (13.0).

### 3.2 Effects of microbial inoculation and planting density on root nodulation

**Figure 2:**
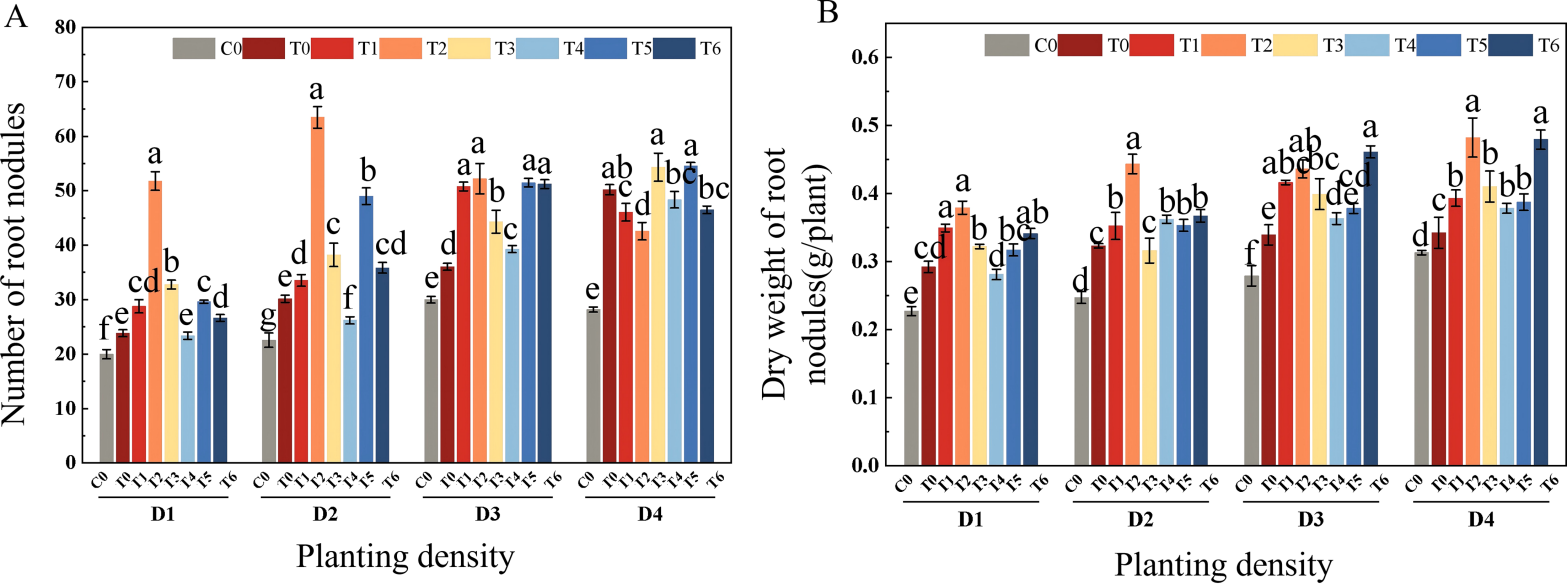
Characteristics of root nodulation in summer-sown soybeans at the podding stage with different microbial inoculants and planting densities. **(A)** Dry weight of root nodules. **(B)** Number of root nodules. All treatments are defined in **Figure 1** legend. Error bars represent standard deviation of the mean (*n =* 5), and significant differences among the treatment groups are marked by different lowercase letters above the error bars (*p* < 0.05).

The dry weight and number of root nodules trended lower with increasing planting density, and both variables significantly responded to microbial inoculation (**Figure 2**). The highest values were observed in the T2 treatment, indicating substantially enhanced nodulation. Nodule dry weight varied with different microbial inoculants (T2 > T6 > T1 > T3 > T5 > T4 > T0 > C0), and the peak value was found in D4T2 (0.483 g) among all treatments. Nodule number increased with higher planting densities in T1 and T3, whereas in other treatments there was an increase followed by a decrease. Plants of the T2 treatment had superior nodulation potential, which was not affected by planting density and improved by 12.1–52.0% over that of other treatments. The D2T2 treatment achieved the largest nodule number (64.00).

### 3.3 Plant dry matter accumulation influenced by microbial inoculation and planting density

**Figure 3:**
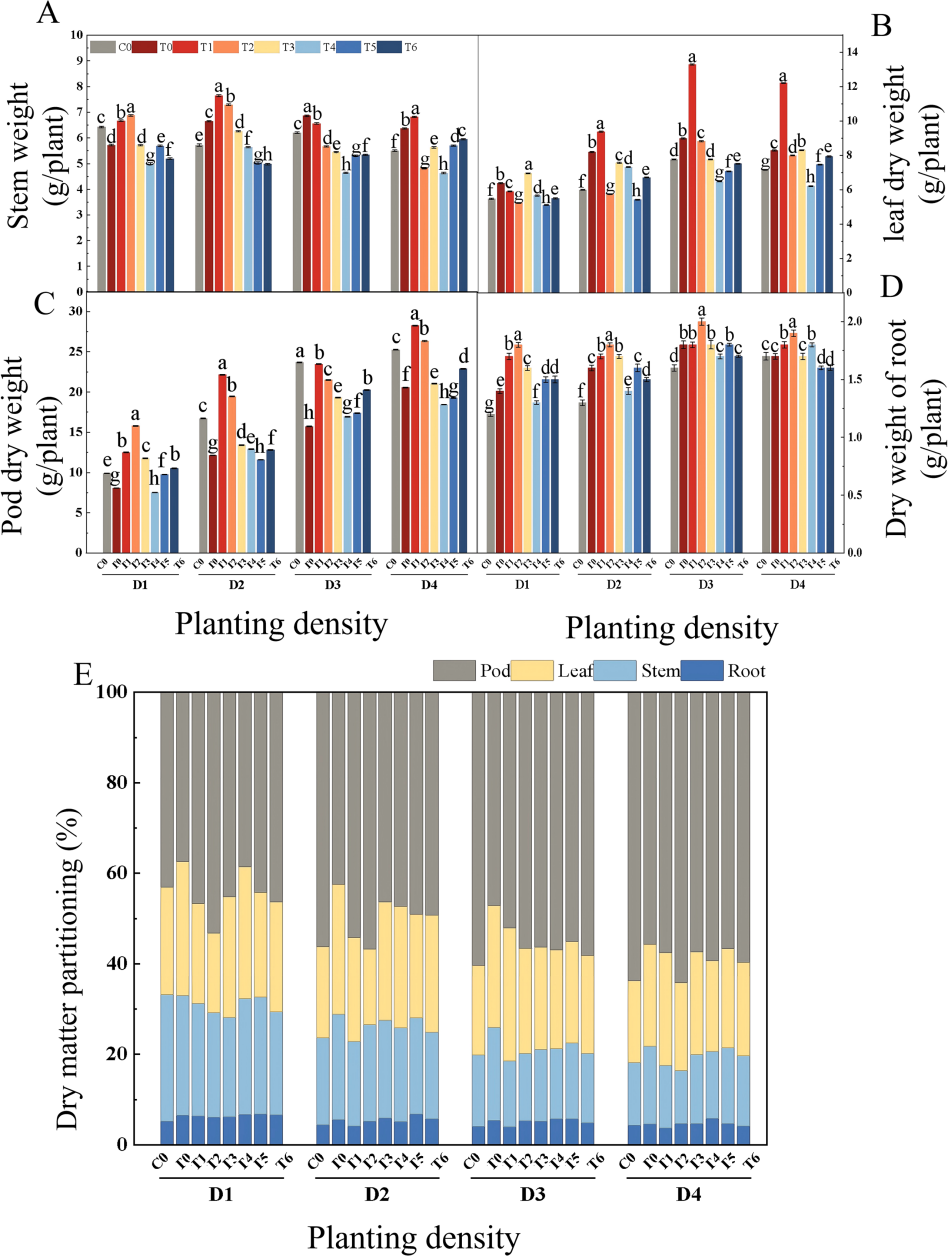
Dry matter accumulation and distribution in summer-sown soybeans at the podding stage with different microbial inoculants and planting densities. **(A)** Stem weight. **(B)** Leaf dry weight. **(C)** Pod dry weight. **(D)** Root dry weight. **(E)** Dry matter partitioning. All treatments are defined in **Figure 1** legend. Error bars represent standard deviation of the mean (*n =* 5), and significant differences among the treatment groups are marked by different lowercase letters above the error bars (*p* < 0.05).

Under the D2 density, the T1 and T2 treatments led to higher stem dry matter accumulation (7.64 g and 7.29 g, respectively) (**Figure 3**). Under the D3 density, the T1 treatment resulted in higher leaf dry matter accumulation (13.28 g), while the T2 treatment contributed to the highest root dry matter accumulation (2 g). Under the D4 density, higher pod dry matter accumulation occurred in the T1 treatment (28.26 g). The proportion of pod dry matter accumulation decreased in response to increased planting densities. Microbial co-inoculants improved the proportion of pod dry matter accumulation compared to single-strain inoculants. A relatively high proportion of pod dry matter accumulation was obtained in the T2 treatment.

### 3.4 Plant nutrient accumulation under microbial inoculation and planting density

**Figure 4:**
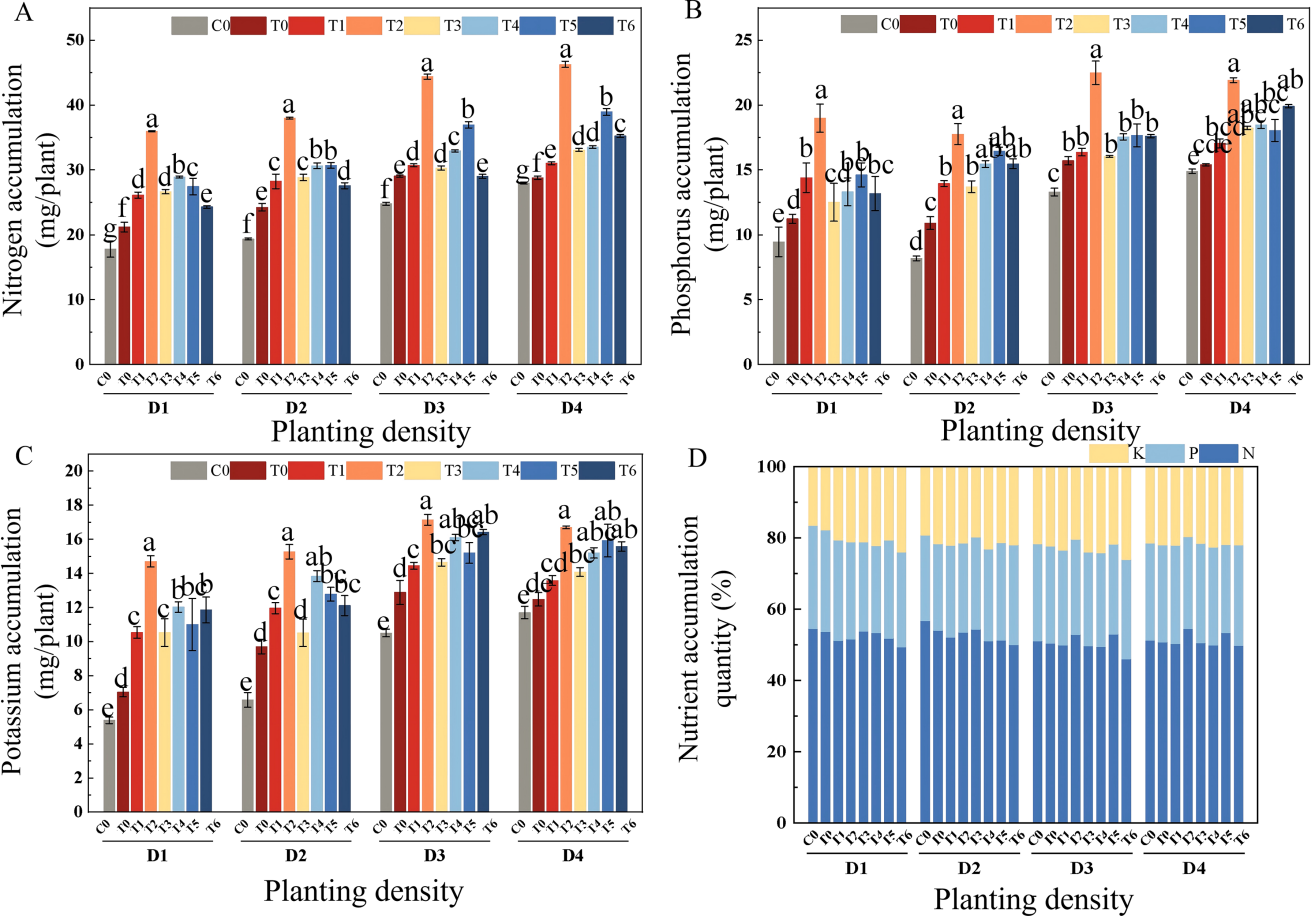
Nutrient accumulation and distribution in summer-sown soybeans with different microbial inoculants and planting densities. **(A)** Nitrogen accumulation. **(B)** Phosphorus accumulation. **(C)** Potassium accumulation. **(D)** Nutrient distribution ratio. All treatments are defined in **Figure 1** legend. Error bars represent standard deviation of the mean (*n =* 5), and significant differences among the treatment groups are marked by different lowercase letters above the error bars (*p* < 0.05).

High nitrogen accumulation was induced in roots when the T2 treatment was applied under the D4 density (**Figure 4**). With increasing planting density, nitrogen, phosphorus, and potassium accumulation in the roots exhibited a downward trend. Under the same planting densities, nutrient accumulation was enhanced in T2 compared to other treatments, and the lowest level was observed in the C0 treatment. Nitrogen accumulation peaked in the D4T2 treatment; the same level was reached in the D3T2 treatment, alongside the highest phosphorus and potassium accumulation. Regarding nutrient distribution, inoculated plants (T1–T6) under relatively high densities (D1, D2) showed a lower proportion of nitrogen accumulation than controls (C0). Among them, the T6 treatment was the worst for nitrogen distribution. Under relatively low densities (D3, D4), the proportion of nitrogen accumulation in T2 was higher than that of other treatments; the peak value occurred in the D4T2 treatment, followed by the D3T2 treatment. However, the T6 treatment resulted in a peak of potassium accumulation across all planting densities, and the overall performance was highest under the D3 density.

### 3.5 Impacts of microbial inoculation and planting density on soybean yield

Various microbial inoculants and planting densities exhibited distinct effects on the yield and yield components of soybeans across the two years (**Tables 3** and **4**). Single-strain inoculants (T0, T4, T5, T6) did not increase soybean yield, pod number per plant, or seed number per plant under the same planting densities. Nevertheless, microbial co-inoculants significantly increased seed number per plant in the T2 treatment and pod number per plant in the T1 treatment followed by the T2 treatment. When the same microbial inoculants were used, pod number per plant, seed number per plant, and 100-seed weight decreased with higher planting densities (D4 > D3 > D2 > D1). The yield first increased and then decreased with increasing planting density (D3 > D4 > D2 > D1). In both years of the experiment, pod number per plant was highest in the T4 treatment under D4 density (36.33 and 36.06). Seed number per plant peaked in the T2 treatment under the D4 density (84.48 and 75.53). The highest yield was observed in the T2 treatment under the D3 density (5174.6 kg hm^–2^ and 5385.6 kg hm^–2^).

**Table 3:** Effects of different treatments on yield and yield components of summer-sown soybean in 2021.

Treatments	Pod number	Seed number	100-seed weight (g)	Yield (kg hm^–2^)
Density (D)				
D1	12.84d	29.63d	14.28d	3655.45d
D2	19.57c	41.82c	14.59c	4011.85c
D3	25.45b	63.28b	15.08b	4494.25a
D4	31.11a	73.80a	16.02a	4219.40b
Inoculant (T)				
C0	19.13e	45.08h	14.68b	3465.46g
T0	17.54f	46.28g	14.99b	3718.84f
T1	24.88a	53.11d	15.05b	4276.71c
T2	24.46b	60.00a	14.85b	4591.56a
T3	23.58c	56.71b	14.87b	4307.6bc
T4	22.46d	49.71f	15.10b	4341.29b
T5	22.29d	53.59c	15.50a	3904.23e
T6	23.59c	52.57e	14.92b	4156.22d
Interaction (D×T)				
D1C0	9.33u	23.25w	14.11klm	3067.5q
D1T0	10.08t	26.73v	14.15jklm	3256.23p
D1T1	12.67s	29.60u	14.33ijklm	3773.6lmn
D1T2	15.67o	33.77r	14.53hijklm	4127.38j
D1T3	14.33p	32.69s	13.75m	3809.57lm
D1T4	12.96rs	26.91v	14.38ijklm	4007.07k
D1T5	14.23pq	32.84s	14.97efghij	3368.54o
D1T6	13.46qr	31.26t	14.00lm	3833.74l
D2C0	18.33m	35.56q	13.98lm	3290.52p
D2T0	14.07pq	34.94q	14.23jklm	3332.09op
D2T1	22.30k	44.23o	14.82fghijkl	4321.61gh
D2T2	22.18k	45.30m	14.67ghijkl	4396.17efg
D2T3	16.67n	45.12mn	14.71ghijkl	4368.26efg
D2T4	23.88j	39.82p	14.62ghijkl	4322.85gh
D2T5	19.66l	44.33no	14.96efghijk	3715.41n
D2T6	19.44l	45.25m	14.77fghijkl	4347.87fgh
D3C0	21.67k	54.52l	14.91efghijk	3741.42mn
D3T0	19.67l	54.82l	15.6bcdef	4242.27i
D3T1	30.23e	61.42j	15.12efghi	4582.23d
D3T2	28.33f	76.45c	14.81fghijkl	5174.59a
D3T3	27.00gh	71.63e	14.75ghijkl	4771.20b
D3T4	24.87i	59.80k	15.26defgh	4634.84cd
D3T5	23.49j	62.96i	15.32cdefgh	4372.93efg
D3T6	28.30f	64.63h	14.88efghijk	4434.54e
D4C0	27.17g	67.00g	15.7bcde	3762.4lmn
D4T0	26.33h	68.62f	15.99abcd	4044.76k
D4T1	34.33b	77.18bc	15.93bcd	4429.38ef
D4T2	31.67d	84.48a	15.38cdefg	4668.09c
D4T3	36.33a	77.41b	16.28ab	4281.38hi
D4T4	28.13f	72.33e	16.13abc	4400.4efg
D4T5	31.80d	74.26d	16.74a	4160.05j
D4T6	33.15c	69.15f	16.03abcd	4008.76k

All treatments are defined in **Table 1** footnote.

Data are presented as mean values (*n =* 5), and different lowercase letters in a column denote significant differences among the treatment groups (*p* < 0.05).

**Table 4:** Effects of different treatments on yield and yield components of summer-sown soybeans in 2022.

Treatment	Pod number	Seed number	100-seed weight (g)	Yield (kg hm^–2^)
Density (D)				
D1	13.62d	28.12d	15.52d	3747.8d
D2	17.49c	39.76c	15.96c	4163.04c
D3	22.83b	52.74b	16.65b	4570.22a
D4	29.31a	63.89a	17.36a	4391.66b
Inoculant (T)				
C0	15.49g	35.74h	16.15cd	3420.32h
T0	18.69f	43.03g	16.23bcd	3897.94f
T1	20.62d	46.49d	16.56abc	4328.98d
T2	23.01c	53.81a	16.45abcd	4965.43a
T3	25.34a	50.84b	16.11d	4243.85e
T4	19.01f	44.04f	16.61ab	4618.02b
T5	19.88e	45.79e	16.82a	3766.30g
T6	24.45b	49.32c	16.06d	4504.59c
Interaction (D×T)				
D1C0	9.91s	22.20s	15.23k	3169.29u
D1T0	13.63p	27.14r	15.53hijk	3368.36s
D1T1	12.50q	28.68q	15.81fghijk	3919.24no
D1T2	15.69no	33.92n	15.78ghijk	4548.64gh
D1T3	15.90n	30.67p	15.02k	3794.42q
D1T4	11.68r	21.13s	15.70ghijk	4139.27lm
D1T5	12.78q	29.15q	15.79ghijk	3119.64u
D1T6	16.87m	32.10o	15.28k	3923.53n
D2C0	10.33s	31.64op	15.33jk	3249.93t
D2T0	15.96n	34.84n	15.75ghijk	3838.12pq
D2T1	19.67k	41.94l	16.36cdefgh	4379.13i
D2T2	20.66j	47.18k	16.25defghi	4931.95cd
D2T3	17.18m	49.07j	15.89fghijk	4076.05m
D2T4	20.05jk	43.21l	16.18efghij	5031.77b
D2T5	15.87n	33.61n	16.5cdefg	3539.62r
D2T6	20.17jk	36.63m	15.47ijk	4257.77j
D3C0	15.03o	36.38m	16.87bcde	3417.63s
D3T0	18.09l	50.09ij	16.69bcdef	4477.72h
D3T1	21.95i	50.88i	16.51cdefg	4401.24i
D3T2	25.77g	58.59g	16.67bcdef	5385.67a
D3T3	32.23b	60.85ef	16.4cdefgh	4902.80d
D3T4	19.36k	50.62i	17.05bcde	4729.91f
D3T5	20.66j	53.06h	16.68bcdef	4235.84jk
D3T6	29.52d	61.45e	16.38cdefgh	5010.92b
D4C0	26.68f	52.73h	17.18bc	3844.43opq
D4T0	27.08f	60.03f	16.94bcde	3907.57nop
D4T1	28.36e	64.44c	17.55b	4616.32g
D4T2	29.9d	75.53a	17.11bcd	4995.44bc
D4T3	36.06a	62.77d	17.12bcd	4202.13jkl
D4T4	24.94h	61.19ef	17.53b	4571.15g
D4T5	30.20d	67.37b	18.33a	4170.09kl
D4T6	31.23c	67.10b	17.12bcd	4826.13e

All treatments are defined in **Table 1** footnote.

Data are presented as mean values (*n =* 5), and different lowercase letters in a column denote significant differences among the treatment groups (*p* < 0.05).

### 3.6 Factors influencing soybean yield

**Figure 5:**
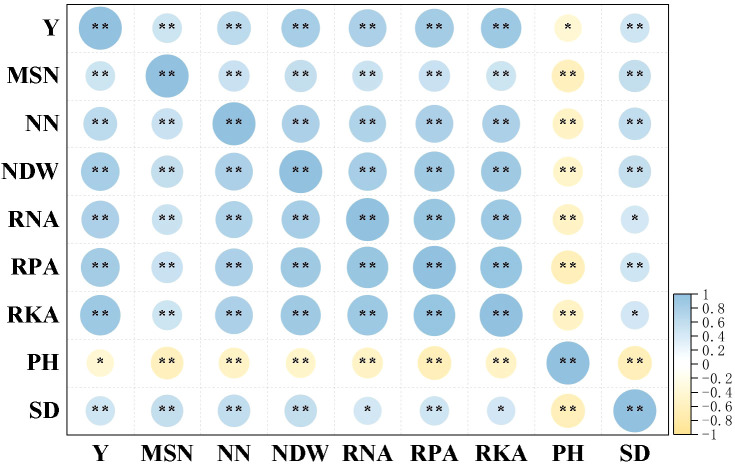
Factors influencing soybean growth, development, and yield under different treatments of microbial inoculation and planting density. **(A)** Correlation heatmap showing the relationships among plant variables. Y, yield; MSN, number of main-stem nodes; NN, number of root nodules; NDW, dry weight of root nodules; RNA, Nitrogen accumulation; RPA, Phosphorus accumulation; RKA, Potassium accumulation; PH, plant height; SD, stem diameter. *p < 0.05, **p < 0.01, and ***p < 0.001.

There were very strong positive correlations between stem diameter, main-stem node number, root nodule number, nodule dry weight, and root nutrient accumulation, all of which were negatively correlated with plant height (**Figure 5A**). Specifically, main-stem node number was positively correlated with stem diameter, and both variables were negatively correlated with plant height (*r* = 0.563 and -0.590, respectively). Strong positive correlations emerged between nodule dry weight and root nitrogen, phosphorus, and potassium accumulation (*r* = 0.800, 0.878, 0.875) This indicates that appropriate microbial inoculation and planting density promoted root growth, development, and nutrient accumulation, thereby increasing the dry weight of the underground part. Both nodule dry weight and root nutrient accumulation were negatively correlated with plant height (*r* = -0.488, respectively). Yield had highly significant positive correlations with stem diameter (*r* = 0.471), main-stem node number (*r* = 0.482), root nodule number (*r =* 0.625), nodule dry weight (*r =* 0.806), and root nutrient accumulation (*r =* 0.776, 0.823, and 0.881). These results indicate close relationships of plant variables under the interaction of microbial inoculation and planting density.

Based on the results of correlation analysis, five variables were selected for comprehensive evaluation by PCA (**Tables 5**–**7**).The principle of principal component analysis mainly involves reducing multiple variables to a few by means of dimensionality reduction, thereby enhancing the independence among indicators. When conducting the analysis, the rule of eigenvalue > 1 is the most favorable for extracting principal components. As shown in **Table 5**, two principal components were extracted. The eigenvalue of the first principal component was 3.40877, with a variance percentage of 68.2%; the eigenvalue of the second principal component was 0.86496 (close to 1), with a variance percentage of 17.3%. Together, they accounted for 85.5%, indicating that the two extracted principal components encompassed 85.5% of the information in the original data. Through the principal component analysis method for comprehensive evaluation, The first PC integrated yield, root nutrient accumulation, nodule number, and nodule dry weight, while the second PC represented the number of main-stem nodes. The comprehensive score (F) was obtained based on the score of each PC and corresponding variance contribution (F = F1 × 68.2% + F2 × 17.3%). A higher F score suggests a stronger contribution to plant growth, supporting high yield. The results showed that the T0–T6 treatments had different contributions to soybean growth (**Table 7**).The highest F score was obtained in the T2 treatment under the D3 density (1.28), and the effect of planting density was ranked as: D3 > D4 > D2 > D1. The result indicates that the D3T2 treatment was optimal for achieving high-yield potential in soybeans.

**Table 5:** Total variance explained.

Principal component number	Initial eigenvalues			Extract the sum of squares of the loads		
	Aggregate	Percentage of variance (%)	Cumulative (%)	Aggregate	Percentage of variance (%)	Cumulative (%)
1	3.40877	68.1755	68.1755	3.40877	68.1755	68.1755
2	0.86496	17.29918	85.47468	0.86496	17.29918	85.47468
3	0.38692	7.73838	93.21306			
4	0.18338	3.66767	96.88073			
5	0.15596	3.11927	100			

**Table 6:** Principal component analysis table.

Metric	Principal component	
	PC1	PC2
Y	0.46791	-0.19426
MSN	0.23851	0.9612
RNAc	0.50175	-0.17206
NN	0.47036	-0.01178
NDW	0.50118	-0.09276
aggregate	3.40877	0.86496
Percentage of Variance (%)	68.1755	17.29918
Cumulative (%)	68.1755	85.47468

**Table 7:** Comprehensive scoring and ranking of different treatments for the yield of summer-sown soybeans.

Treatment	Principal component score		Comprehensive score (F)	Comprehensive score ranking
	PC1 (F1)	PC2 (F2)		
D1C0	-1.94	1.62	-1.04	30
D1T0	-1.42	1.42	-0.72	27
D1T1	-0.59	0.36	-0.34	22
D1T2	0.85	1.20	0.79	6
D1T3	-0.67	0.45	-0.38	23
D1T4	-1.16	-2.63	-1.25	32
D1T5	-1.11	-0.95	-0.92	28
D1T6	-0.96	-1.89	-0.98	29
D2C0	-1.80	1.02	-1.05	31
D2T0	-0.86	1.16	-0.39	24
D2T1	-0.17	0.11	-0.10	18
D2T2	1.55	0.92	1.22	2
D2T3	-0.34	-0.01	-0.23	21
D2T4	0.02	-0.19	-0.02	17
D2T5	-0.20	-0.62	-0.20	20
D2T6	-0.11	-0.53	-0.17	19
D3C0	-1.01	0.87	-0.54	25
D3T0	-0.06	0.31	0.02	16
D3T1	0.67	-0.48	0.37	10
D3T2	1.83	0.22	1.28	1
D3T3	0.57	-0.85	0.24	11
D3T4	0.53	0.40	0.43	9
D3T5	0.53	-1.35	0.13	12
D3T6	1.16	0.42	0.86	5
D4C0	-0.84	-0.73	-0.70	26
D4T0	0.03	0.22	0.06	15
D4T1	0.42	-0.98	0.12	13
D4T2	1.59	0.07	1.10	3
D4T3	0.55	-1.52	0.11	14
D4T4	0.75	0.74	0.64	8
D4T5	0.90	0.56	0.71	7
D4T6	1.20	0.64	0.93	4

All treatments are defined in **Table 1** footnote.

## 4 Discussion

### 4.1 Microbial inoculation and planting density modulate plant growth and development in summer-sown soybeans

Symbiotic nitrogen fixation between soybean and rhizobia plays a vital role in plant growth and development. The efficiency of symbiotic nitrogen fixation is associated with a range of factors, such as the compatibility between rhizobia and soybean varieties, the nodulation and nitrogen-fixing capacity of rhizobia, and the ambient environment (e.g., soil status, moisture level, temperature) (Sharaf et al., 2019). Compound microbial fertilizers contain abundant humus, essential nutrients, and beneficial microorganisms, which can effectively promote plant growth (Liu et al., 2020). In this study, we evaluated how microbial inoculation and planting density interact to modulate the vegetative growth of summer-sown soybeans in the arid region of southern Xinjiang. This evaluation represents a key component of testing our hypothesis that integrated agronomic practices can optimize soybean population structure for high yield.

Two-year experimental results demonstrated the accelerated growth rates of plant height in summer-sown soybeans during the V4–R4 growth stages. This was followed by decelerated growth rates of plant height after the R6 stage, with consistent growth trends across all treatments. Soybean stem diameter increased continuously as the growth period progressed. Wilcox et al. (1981) have reported that soybean plant height increases while stem diameter decreases with increasing planting density. This is due to the formation of a low-light microenvironment within high-density soybean populations, which triggers a shading response characterized by increased plant height, thinner stems, and reduced main-stem node number. In contrast, we found that microbial inoculation suppressed excessive plant growth in soybeans under increased planting densities, as indicated by reduced plant height and decreased number of main-stem nodes (Postma et al., 2021; Ruberti et al., 2012).

Dalvan do Nascimento et al. (2022) observed that soybeans inoculated with *Bacillus* exhibited superior plant height growth compared to non-inoculated controls. Many *Bacillus* species produce hormones, enzymes, antibiotics, and siderophores that enhance plant growth and confer stress tolerance (Abuhena et al., 2024). Our results revealed that soybean plant height was highest in the co-inoculation treatment of T3 under the high planting density of D1 (62.62 cm). The largest stem diameter (0.697 cm) and the greatest number of main-stem nodes (13 nodes) were respectively observed in the co-inoculation treatments of T1 and T2 under the medium planting density of D3. These results indicate that compared to single inoculation with *Bacillus*, co-inoculation with rhizobia effectively improved plant growth and development. However, the effects of different microbial co-inoculants varied depending on rhizobial species and planting density. Therefore, soybean cultivation should take into full account the selection of microbial inoculant and the adjustment of planting density to lay a solid foundation for high-yield production.

### 4.2 Microbial inoculation and planting density affect root nodulation in summer-sown soybeans

Nitrogen is one of the three major essential nutrients playing a crucial role in crop growth and development (Hu and Chu, 2020). For every 1, 000 tonnes of soybean seeds produced, approximately 65 tonnes of pure nitrogen is absorbed from the external environment. Nitrogen fixation by root nodules can only supply 40–60% of the total nitrogen required by soybean plants (Donahue et al., 2020). Thus, relying solely on nitrogen fixation by root nodules cannot meet the nitrogen demand of soybeans during the vigorous growth period.

*Bacillus* spp. are commonly used as microbial inoculants (Kaschuk et al., 2016; Ke et al., 2022). These PGPR have been extensively studied for their plant growth-promoting effects and functional gene mechanisms (Jiang et al., 2025; Miftakhurrohmat and Sutarman, 2021). We showed that co-inoculation of summer-sown soybeans with *Bacillus* and rhizobia markedly increased root nodule number and root dry weight. *Bacillus* spp. have been found to promote nodulation in soybeans by producing indole-3-lactic acid (Qiu et al., 2025). However, high-density planting restricted the growth and formation of soybean root nodules. Increased planting density likely inhibited rhizobial infection of root hairs, which reduced nodule number during initial nodulation and impaired the efficiency of nodule nitrogen fixation (Lu et al., 2024).

As planting density increases, intraspecific competition patterns are expected to be reorganized (Lekberg et al., 2018). As such, plants adjust their resource acquisition strategies through active or passive physiological adaptations (Guo et al., 2024). We found that the nodule number per plant increased with increasing planting density. Upon co-inoculation of *Bacillus* and rhizobia, the nodule number first increased and then decreased with higher planting densities. Although this phenotypic adjustment reduced nodule number, it increased nodule dry weight per plant. For example, nodule dry weight increased by 25% under the highest planting density compared to the lowest density. Denser planting potentially promoted more vigorous root growth, thereby improving the transmission and colonization efficiency of microbial inoculants in soybean roots.

### 4.3 Microbial inoculation and planting density influence dry matter accumulation and distribution in summer-sown soybeans

Total dry matter accumulation is the foundation of soybean yield formation and a key agronomic trait reflecting yield potential (Raza et al., 2024; Yuan et al., 2011). Dry matter accumulation during soybean growth positively correlates with final yield (Qin et al., 2025). Consistent with the optimal allocation theory, soybean dynamically redistributes dry matter among organs under high-density conditions, and this crop prioritizes resource allocation to organs that acquire limiting resources (Lemaire et al., 2019). Our results showed that plant dry matter accumulation gradually increased with the reproductive development of summer-sown soybeans. The dry matter accumulation in various organs initially increased and then decreased, reaching a maximum at the R6 stage across all treatments.

Javier De Luca et al. (2014) have reported that soybean adapts to high-density environments by reducing leaf number and area. We also found that under high planting density, biomass allocation to soybean leaves decreased rapidly during the seed-filling stage, while more resources were allocated to the roots and pods. Microbial co-inoculation considerably affected the dry weight of soybean organs. Planting density primarily influenced soybean yield through its impact on dry matter accumulation and distribution during the mid-to-late growth stages (An et al., 2025). Bacterial inoculation did not alter the effect of planting density on dry matter yield. Nevertheless, dry matter yield was increased across all density levels upon inoculation, and more pronounced yield differences were induced under high-density conditions.

At the pod-filling stage, the total dry matter accumulation of soybeans and dry matter translocation to seeds varied among treatments. Stem dry matter accumulation initially increased and then decreased with rising planting density, while pod dry matter accumulation exhibited the opposite trend. These patterns may be attributed to limited photosynthetic nutrient accumulation and intense competition for soil resources among soybean plants under high-density planting. Under the same planting densities, the T2 treatment achieved the highest proportion of pod dry weight (53.2–64.1%), which was much higher than that of the T1 treatment. This disparity highlights the advantage of *Rhizobium* SN7–2 over *Rhizobium* SMH12 in regulating dry matter distribution towards soybean pods when co-inoculated with *Bacillus*.

### 4.4 Microbial inoculation and planting density dictate root nutrient absorption in summer-sown soybeans

Nitrogen, phosphorus, and potassium are the most abundant nutrients in soybeans and these elements play key roles in plant growth, photosynthesis, and yield formation (Wiggans, 1939). Inoculation with *Bacillus* and rhizobia can boost plant growth (Miljaković et al., 2022). The accumulation of these nutrients in crops is crucial for yield. Numerous studies have indicated that the accumulation patterns of nutrients in crops mirror that of dry matter, as manifested by pronounced positive correlations between them (Wei et al., 2020; Zhou et al., 2025). Luo et al. (2025) observed similar trends in the dry matter and nitrogen accumulation of soybeans. These findings are in agreement with our results from the two-year experiment.

The root system is a critical organ for plant growth and stress resistance (Kirschner et al., 2021). We observed that nutrient accumulation in soybean roots trended lower with increasing planting density. On the one hand, insufficient fertilizer supply under high-density planting may reduce nitrogen assimilation, translocation efficiency, and translocation to seeds during the late growth stages of soybean. On the other hand, excessive fertilizer input under low-density planting can accelerate vegetative organ development and dry matter accumulation, while reducing nutrient translocation to seeds (An et al., 2025).

Rajendran et al. (2008) have shown that inoculating rhizobia and *Bacillus* can promote plant growth. Our results indicated that T2 was the optimal treatment for enhancing nutrient accumulation in soybeans under relatively low densities (D3, D4). This can be attributed to *Bacillus-*mediated improvement of rhizobial infection and nodule organogenesis, which contributes to nitrogen supply. A certain nitrogen level can enhance dry matter and nitrogen accumulation in soybeans, especially during the podding and filling stages. In the early growth stages, the nitrogen content in leaves increases notably. As the growth period progresses, dry matter accumulates in each organ and reaches its peak after the podding and filling stages; then, dry matter is translocated to the pod wall and seed parts (Pannecoucque et al., 2022; An et al., 2025).

Crops are highly sensitive to potassium deficiency, which can have a significant impact on biomass accumulation, yield and quality (Sale and Campbell, 1986; Gerardeaux et al., 2009; Ma et al., 2013). Previous studies on cotton have shown that under moderate potassium deficiency, leaf area and dry matter yield decline significantly, but the photosynthetic rate of leaves or the ground canopy does not decrease significantly (Reddy and Zhao, 2005). Therefore, unlike nitrogen or phosphorus, potassium deficiency seems to mainly affect the supply and transport of sugars, metabolites and other minerals between plant organs. Potassium plays an important role in promoting growth, reducing the impact of adverse conditions, increasing yield and improving product quality (including shelf life, market value and economic value). Relevant studies on tomato plants have shown that increasing the application rate of potassium fertilizer significantly improves fruit quality, with vitamin C being positively correlated with leaf potassium concentration, suggesting that potassium may help improve fruit quality (Liu et al., 2008). In leguminous plants, potassium, as the most abundant cation in cells, plays an important role in the formation of strong root systems and their water absorption, as well as root hair growth, which in turn promotes the formation of root nodules and biological nitrogen fixation (Nakei et al., 2022). This is consistent with the results of this study, where the potassium content and nodule traits in the T2 treatment were the best.

### 4.5 Microbial inoculation and planting density determine the yield of summer-sown soybeans

This study revealed that inoculation with *Bacillus* and/or rhizobia enhanced the yield of summer-sown soybeans. Several microbial co-inoculants proved more effective than single-strain inoculants in increasing the numbers of pods and seeds per plant. Under reduced planting density, the T3 treatment was conducive to increasing the number of pods per plant of soybean, while the T2 treatment was favorable for increasing the number of seeds per plant. This indicates that various rhizobial strains have distinct physiological and ecological characteristics, which may affect their symbiotic relationship with soybeans and their effects on plant growth.

In agricultural production, inoculating *Bacillus* and rhizobia can soybean yield by increasing pod and seed numbers per plant (Gan et al., 2010; Ngosong et al., 2022). We found that under the same planting densities, microbial inoculation increased root nitrogen, phosphorus, and potassium contents, in addition to enhancing nodule nitrogen-fixing capacity soybeans. Inoculated plants provided more energy to aboveground organs, ultimately improving soybean yield. Ran et al. (2023) showed that 100-seed weight, seeds per plant, and pods per plant all decreased with increasing planting density of the soybean cultivar SN35 under field conditions. In contrast, we found that upon inoculation with *Bacillus* and rhizobia, soybean yield initially rose and then declined with increasing planting density. This synergistic effect was particularly evident in the T2 treatment, as manifested by vigorous vegetative growth (increased main-stem nodes and underground dry matter weight), alongside improved growth efficiency (increased seeds per plant, 100-seed weight, and yield per plant).

In the two-year experiment, the highest soybean yield (5, 174.6–5, 385.6 kg·hm^–2^) was observed when *Bacillus* was co-inoculated with *Rhizobium* SN7-2 (T2) under a planting density of 590, 000 plants hm^–2^ (D3). The D3T2 treatment achieved a 38.3–57.6% yield increase over the control treatment under the same planting density. The dual approach fosters stable yield growth in soybeans by reducing interplant stress under high-density conditions and compensating for weakened plant competition under low-density conditions. Furthermore, integrated agronomic practices create a favorable agroecosystem that coordinates nutrient supply, underground growth, and overall plant balance. This ultimately contributes to pod filling, seed weight, and yield potential in soybeans (Guo et al., 2024; Yamamoto et al., 2025; Zhao et al., 2018).

## 5 Conclusion

This study uncovered that microbial inoculation and planting density played a synergistic role in regulating plant growth, root nodulation, dry matter and nutrient distribution, and yield of summer-sown soybeans in southern Xinjiang. High-density planting (D1) increased plant height while reducing stem diameter and the number of main-stem nodes. Co-inoculation with *Bacillus* and *Rhizobium* SN7-2 (T2) mitigated the inhibition of stem diameter growth caused by high planting density and increased the node number under medium to low densities (D3, D4), thereby optimizing plant morphology. In the T2 treatment, the number and dry weight of root nodules notably increased, showing the greatest nodulation potential. This effect was more pronounced under medium planting densities (D2, D3), allowing for efficient nitrogen fixation by the roots. The T2 treatment promoted dry matter accumulation in the pods and optimized nutrient distribution in the roots. Nitrogen, phosphorus, and potassium accumulated to relatively high levels in the plants under the D3 density. The T2 treatment particularly increased the number of seeds per plant, and the yield reached its peak under the D3 density across two years (5, 174.6 kg hm^–2^ and 5, 385.6 kg hm^–2^). Main-stem node number, root dry matter weight, and root nitrogen accumulation were identified as the key factors influencing yield, and the D3T2 treatment contributed the most to plant growth. Under the experimental conditions, co-inoculation of soybeans with *Bacillus* and *Rhizobium* SN7-2 (T2) at a planting density of 590, 000 plants hm^–2^ (D3) represents the optimal practice for achieving high-yield cultivation. This integrated strategy improves soybean yield through multifaceted effects, including promotion of plant growth, enhancement of root nodulation and nitrogen fixation, and optimization of dry matter and nutrient distribution.

## Data Availability

The original contributions presented in the study are included in the article/supplementary material. Further inquiries can be directed to the corresponding authors.
